# Comparison of Clinical and Parasitological Data from Controlled Human Malaria Infection Trials

**DOI:** 10.1371/journal.pone.0038434

**Published:** 2012-06-11

**Authors:** Meta Roestenberg, Geraldine A. O'Hara, Christopher J. A. Duncan, Judith E. Epstein, Nick J. Edwards, Anja Scholzen, André J. A. M. van der Ven, Cornelus C. Hermsen, Adrian V. S. Hill, Robert W. Sauerwein

**Affiliations:** 1 Department of Medical Microbiology, Radboud University Nijmegen Medical Center, Nijmegen, The Netherlands; 2 Centre for Clinical Vaccinology and Tropical Medicine and Jenner Institute, Churchill Hospital University of Oxford, Oxford, United Kingdom; 3 Department of General Internal Medicine, Radboud University Nijmegen Medical Center, Nijmegen, The Netherlands; 4 U.S. Military Malaria Vaccine Program, Naval Medical Research Center, Silver Spring, Maryland, United States of America; World Health Organization, Switzerland

## Abstract

**Background:**

Exposing healthy human volunteers to *Plasmodium falciparum*-infected mosquitoes is an accepted tool to evaluate preliminary efficacy of malaria vaccines. To accommodate the demand of the malaria vaccine pipeline, controlled infections are carried out in an increasing number of centers worldwide. We assessed their safety and reproducibility.

**Methods:**

We reviewed safety and parasitological data from 128 malaria-naïve subjects participating in controlled malaria infection trials conducted at the University of Oxford, UK, and the Radboud University Nijmegen Medical Center, The Netherlands. Results were compared to a report from the US Military Malaria Vaccine Program.

**Results:**

We show that controlled human malaria infection trials are safe and demonstrate a consistent safety profile with minor differences in the frequencies of arthralgia, fatigue, chills and fever between institutions. But prepatent periods show significant variation. Detailed analysis of Q-PCR data reveals highly synchronous blood stage parasite growth and multiplication rates.

**Conclusions:**

Procedural differences can lead to some variation in safety profile and parasite kinetics between institutions. Further harmonization and standardization of protocols will be useful for wider adoption of these cost-effective small-scale efficacy trials. Nevertheless, parasite growth rates are highly reproducible, illustrating the robustness of controlled infections as a valid tool for malaria vaccine development.

## Introduction

Deliberate exposure of healthy human volunteers to the bites of laboratory-reared *Plasmodium falciparum* (*Pf)-*infected mosquitoes in a controlled experimental setting is an accepted tool in malaria vaccine development. Such controlled human malaria infection (CHMI) trials can be used to investigate *Pf* immunology [1] or to provide data on the efficacy of malaria vaccine candidates [2] as a precursor to more costly and logistically challenging Phase IIb field efficacy trials. In CHMI, development of blood stage parasites in test subjects is assessed by blood smears at regular time points and anti-malarial treatment is given as soon as blood stage parasites are detected microscopically, keeping blood stage parasitemia low (treatment threshold: four parasites/µl) and confined to a short (two to eight day) period [3]. A comparison of the interval between exposure and parasite detection (prepatent period) among vaccinated and control subjects, together with sterile efficacy rates in vaccinees, provides an important efficacy estimate for the candidate vaccine. Because prepatent periods without information on parasite growth rates provide only an estimate of vaccine efficacy, molecular techniques have been developed to more accurately quantify blood parasites and provide parasite kinetic data [3], [4], [5].

Decades of extensive efforts to find an efficacious malaria vaccine have lead to the development of about 38 *Pf* candidate (sub-unit) malaria vaccines or vaccine components (www.who.int/vaccine_research/links/Rainbow/en/index.html). To meet the demands of the growing malaria vaccine development pipeline, CHMI will likely be conducted in an increasing number of sites worldwide. We have performed a comparative analysis of safety and parasitological data from trials performed at the Radboud University Nijmegen Medical Centre (RUNMC), The Netherlands, and the University of Oxford in the United Kingdom, two of a total of five different institutions and the only non-US institutions currently routinely performing CHMI. Where possible, data were compared with a previously published report from the US Military Malaria Vaccine Program (USMMVP), Naval Medical Research Center Component, Silver Spring, Maryland [6]. Based on this data, we provide a perspective on future strengthening of and improvements to the CHMI model.

## Methods

Volunteers participating in CHMI studies performed at the RUNMC in Nijmegen, The Netherlands and the Centre for Clinical Vaccinology and Tropical Medicine at the University of Oxford, United Kingdom were included from 1999 until 2010 and from 2000 to 2010 respectively. Data were compared with a previously published report of trials performed between 1998 and 2002 at the USMMVP, Silver Spring, Maryland [6].

### Patient population

Data from three different cohorts were assessed. A summary of the cohort characteristics is provided in Table 1. Data from eight studies at RUNMC were analyzed in two cohorts [7], [8], [9], [10]. The first cohort (RUNMC I) has been previously described by Verhage et al [7]. Five volunteers from this cohort received anti-malarial treatment with 48 hours delay after parasites were detected by microscopy. The RUNMC II cohort includes volunteers from 2004 onwards, when more stringent cardiovascular inclusion criteria (based on SCORE cardiovascular risk [11]) were adapted following a case of myocardial infarction in a malaria-negative volunteer [7] and an increased threshold for microscopic parasite detection was implemented. RUNMC II includes 12 volunteers who participated in a candidate malaria vaccine trial, but were not protected (*Nieman et* *al. manuscript in preparation*). All other volunteers were unimmunized.In Oxford, 65 infectivity control volunteers participated in 14 studies [12], [13], [14], [15], [16], [17], [18].

**Table 1:** Cohort characteristics.

Cohort	RUNMC I	RUNMC II	Oxford	USMMVP
Number of volunteers	20	43	65	47
**Demographics**				
Mean age (stdev)	29 (8.3)	22 (2.5)	27 (6.2)	27 (UNK)
Sex (males)	10	14	32	27
Immunized, non-protected volunteers	0	12	0	31
**Methodology**				
Mosquito strain	NF54	NF54	3D7	NF54
Number of infected mosquitoes	4–7	5	5	5
Exposure time to mosq. (min)	10	10	5	5
Threshold microscopy (parasites/ul)	2	4	2	3
Clinical follow-up frequency (times daily)	3	3	2	1
Anti-malarial treatment				
Chloroquine	20		30	47
Artemether/lumefantrine		33	35	
Atovaquone/proguanil		10		
**Parasitological data**				
Median prepatent period (days)	9.0	10.0	11.2	11.0
Range prepatent period (days)	7.3–10.3	7.0–12.3	8.0–14.5	9.0–14.0
Geometric mean peak parasitemia (*Pf*/ml)	7076	15901	9055	
Geometric mean parasitemia first cycle (*Pf*/ml)	567	456	48	
Geometric multiplication factor	11.8	11.1	11.6	
**Laboratory safety parameters**				
Mean platelet count day 7–10 (x10e9/l)	242	261		239
**References**	[7]	[8]–[10]	[11]–[17]	[6]

All included subjects were healthy, male and female, malaria-naïve volunteers between the ages of 18 and 50 years(Table 1). Malaria naiveté was confirmed by medical and travel history. Volunteers from the RUNMC cohorts were also confirmed negative for antibodies against blood-stage Pf by ELISA [19]. Volunteers were excluded in case of known allergies to anti-malarials, pregnancy, systemic disease or chronic use of medication. Volunteers were screened by a physician based on medical history, physical examination, complete blood count, liver and renal function tests, pregnancy test and serological testing for HIV, hepatitis B and C. Volunteers provided written informed consent and all studies were approved by either the RUNMC Committee on Research involving Human Subjects or Central Committee on Research involving Human Subjects (CMO 0004–0090, 0011–0262, 2001/203, 2002/170, 2004/129, 2006/207, NL14715.000.06, NL24193.091.09) or the Oxfordshire Research Ethics Committee or the UK Gene Therapy Advisory Committee (C01.111, C02.069, C02.152, C02.153, C02.266, C02.268, C02.293, C02.305, CL03.100, C03.088, 04/Q1604/93, 06/Q1604/55, 05/Q1604/69, GTAC 160-02).

### Infection procedures

*Anopheles stephensi* mosquitoes were infected with the NF54 strain of *Pf* (RUNMC) or 3D7, a clone originally derived from NF54, (Oxford) following previously described procedures [20]. Both strains are chloroquine sensitive (data not shown).

Fixed numbers of mosquitoes were allowed to bite volunteers during five (Oxford) or ten (RUNMC) minutes. Fully blood-engorged mosquitoes were confirmed positive for salivary gland sporozoites by dissection (a threshold of >10 sporozoites/gland was used in all centers). If necessary, feeding sessions were repeated until exactly the predefined number of infected mosquitoes were fully engorged, i.e. five mosquitoes, except for the RUNMC I cohort, where volunteers were exposed to the bites of four to seven mosquitoes (Table 1). Monitoring took place twice (Oxford) or thrice daily (RUNMC) using microscopy of Giemsa-stained blood smears starting on day five at RUNMC or on the afternoon of day six at Oxford. Volunteers were treated with a standard therapeutic regimen of chloroquine, arthemether/lumefantrine or atovaquone/proguanil as soon as microscopy confirmed the presence of parasites or by the discretion of the physician.

Trial volunteers were followed on an outpatient basis and lived in the vicinity of the hospital. An active tracking policy using mobile phones and/or home visits was operational at both institutions during the monitoring period. Adverse events were recorded at every visit. Investigators evaluated the potential relation of adverse events with trial procedures. All probable or possible related events were included in the analysis, with exception of the five volunteers for whom anti-malarial treatment was delayed by 48 hours. The USMMVP report included adverse events from day seven after challenge [6]. Severity of symptoms in RUNMC II and the USMMVP report were assessed according to standard guidelines (http://www.fda.gov/BiologicsBloodVaccines/GuidanceComplianceRegulatoryInformation/Guidances/Vaccines/ucm074775.htm). Mild symptoms (grade 1) did not interfere with daily activities, moderate symptoms (grade 2) interfered with daily activities, severe symptoms (grade 3) prevented daily activity. Symptom severity was not consistently assessed in the other cohorts, with exception of fever, which was graded mild when 37.5 to 37.9°C, moderate when 38 to 38.9°C and severe when ≥39°C in all cohorts. All centers recorded oral temperatures, which were measured at least once daily, either by volunteers themselves or by the attending physician at the clinical site. RUNMC also recorded auricular temperatures at the clinical site up to three times daily. Serious adverse events (grade 4) were defined according to International Conference of Harmonization Good Clinical Practice Guidelines.

Clinical hematological laboratory data were available on a daily basis from day five post-challenge until three days after anti-malaria treatment for RUNMC cohorts. Biochemical parameters in RUNMC cohorts were assessed once, at three days after anti-malarial treatment. Clinical laboratory parameters in Oxford were not routinely recorded during challenge in any trials. For the USMMVP cohort, clinical laboratory parameters were reported at days 10–12 after challenge.

### Parasitological data

Prepatent period was defined as time from exposure to positive thick smear. The threshold for microscopic detection of parasites varied between cohorts depending on the local standard operating procedure (Table 1). Centres use different microscopes, blood volume and slide surface area. RUNMC and Oxford readers complete 200 fields, yielding a threshold of approximately two parasites per μl blood in RUNMC I and Oxford (slide was deemed positive if one parasite was found), which was confirmed by Q-PCR in Oxford. A threshold of four parasites per μl blood was achieved in RUNMC II (slide was deemed positive if two parasites were found). USMMVP readers completed 5 passes (72 fields/pass), yielding a threshold of approximately 3 parasites per μl blood (slide positive if two parasites were found). In all centers, slides were read by two independent readers at 1000× magnification.

Simultaneously, a quantitative PCR (Q-PCR) for *Pf* was used in Oxford to support microscopy. Parasite densities were measured by Q-PCR for RUNMC and Oxford cohorts as previously described [3], [5]. Although methodology of the Q-PCR differed, there was no inter-institutional difference in measured densities, confirmed by an exchange of samples between both institutions (data not shown). Peak parasitemia was defined as the highest parasite density during infection measured by Q-PCR. Any cycle threshold above 45 was plotted as zero parasitemia. For calculations, these samples were given a value of half the detection threshold (ten parasites/ml).

### Data analysis

Data were assessed in SPSS 16.0 with correction for multiple analyses. Differences between frequencies and prepatent period were compared by Kruskal-Wallis tests when comparing multiple groups or Mann-Whitney U tests when comparing two groups. Dunn's multiple comparisons test was performed as post-hoc analysis when appropriate. Analysis of parasitological PCR data was performed on log-transformed data using independent-samples t-test when comparing two groups and one-way ANOVA when comparing three groups. The multiplication rate of blood stage parasites was calculated by the ratio of the geometric mean parasitemia in the second cycle with the first cycle (day 8.6–10.5 vs 6.6–8.5) or the third cycle with the second cycle (day 10.6–12.5 vs 8.6–10.5), or if possible, the mean of both ratios.

Correlations were assessed by Pearson's correlation when parametric and Spearman's when non-parametric. Two-sided p-values below 0.05 were considered significant unless stated otherwise.

## Results

### Demographics

A total of 128 volunteers were grouped into three different cohorts: RUNMC I and II and Oxford and compared to published data from 47 infected individuals at USMMVP [6], for a total of 175 volunteers. Individuals were generally 20–40 years old, with equal distribution between male and female participants (Table 1). There were no significant differences in sex distribution between the different cohorts (Kruskal-Wallis, p = 0.22), but RUNMC II volunteers were significantly younger (Kruskal-Wallis, p<0.001) due to more stringent cardiovascular inclusion criteria.

### Clinical manifestations

One hundred percent of volunteers developed signs and symptoms of uncomplicated malaria; there was no severe malaria according to WHO criteria (http://whqlibdoc.who.int/publications/2010/9789241547925_eng.pdf). Five serious adverse events (grade 4) occurred; three volunteers were admitted to the hospital for intravenous rehydration and directly observed intake of anti-malarial medication because of vomiting, two from RUNMC II and one from Oxford. These volunteers were discharged within 24–48 hours without sequelae. One volunteer from RUNMC II reported retrosternal chest pain two days after treatment with arthemeter-lumefantrine [21]. She was admitted, diagnosed as acute coronary syndrome and treated accordingly. Apart from acute coronary syndrome, myocarditis can be considered as a final diagnosis. A definite relationship between the cardiac event and malaria could not be established. One volunteer from Oxford was briefly admitted for observation of a suspected allergic reaction to chloroquine that resolved spontaneously and rapidly.

The most frequently reported solicited adverse events were headache, fever and myalgia (Table 2). Approximately 20–50% of volunteers experienced severe (grade 3) adverse events. Arthralgia, fatigue and chills were significantly more frequently reported in Oxford (chills) and USMMVP (arthralgia, fatigue) while fever (temperature >37.5°C) occurred with significant higher frequency in RUNMC and USMMVP.

**Table 2:** Adverse event frequency and duration for three different cohorts.

	RUNMC I ^(12)^ #					RUNMC II ^(10,11)^				USMMVP ^(12)^					Oxford#							
	Frequency			Duration		Frequency		Duration			Frequency		Duration		Frequency		Duration					
Symptom	no		%	mean	SD	no	%	mean	SD		no	%	mean	SD	no	%		mean	SD	Total (%)	p-value*	
headache	19		95	4.74	2.02	40	93	3.75	2.58		47	100	3.75	2.20	61	95		1.30	0.71	96	0.44	
fever	18		90	3.56	3.33	37	86	1.92	0.87		37	79	-	-	20	31		0.62	0.52	64	**<0.001**	
myalgia	13		65	2.92	1.44	32	74	2.86	2.25		38	81	3.00	1.82	48	75		0.58	0.52	75	0.88	
arthragia	-		-	-	-	2	5	2.56	3.45		17	36	2.35	2.29	-	-		-	-	11	**<0.001**	
malaise	13		65	4.69	3.50	28	65	2.16	1.14		44	94	2.61	1.72	-	-		-	-	49	0.01	
fatigue	9		45	3.11	1.54	26	60	4.64	4.36		47	100	3.96	1.69	-	-		-	-	47	**<0.001**	
dizziness	6		30	2.75	2.22	16	37	0.95	2.73		24	51	1.67	0.82	-	-		-	-	26	0.44	
chills	5		25	2.00	0.82	14	33	1.31	0.65		40	85	2.28	1.26	48	75		1.17	0.96	61	**<0.001**	
abdominal pain	2		10	2.33	3.21	12	28	1.62	1.82		17	36	1.82	1.55	-	-		-	-	18	0.25	
nausea	9		45	3.33	2.00	21	49	1.83	1.55		29	62	1.83	1.20	39	61		1.18	0.65	56	0.59	
vomiting	2		10	1.00	0.00	7	16	0.73	0.74		6	13	1.68	0.41	-	-		-	-	9	0.51	
diarrhoea	4		20	2.00	2.71	3	7	0.51	0.57		12	26	1.67	0.89	16	23		0.58	0.52	20	0.15	
cough	4		20	3.80	6.30	2	5	0.05	0.21		9	19	2.57	1.81	-	-		-	-	9	0.08	
**Severe symptoms (grade III)**																						
any		-	-	-	-	21	49	3.00	2.36		10	21	2.30	1.25	-	-		-	-	34	0.01	
headache		-	-	-	-	6	14	5.17	3.55		3	6	2.00	1.00	-	-		-	-	10	0.26	
fever		8	40	-	-	15	35	1.92	0.94		-	-	-	-	-	-		-	-	37	**<0.001**	
myalgia		-	-	-	-	0	0	0.00	0.00		1	3	3.00	0.00	-	-		-	-	1	0.26	
malaise		-	-	-	-	7	16	2.50	1.25		3	7	1.33	0.58	-	-		-	-	11	0.25	
fatigue		-	-	-	-	2	5	7.40	0.57		2	4	3.50	1.12	-	-		-	-	4	0.70	
nausea		-	-	-	-	0	0	0.00	0.00		1	3	1.00	0.00	-	-		-	-	1	**<0.001**	
chills		-	-	-	-	0	0	0.00	0.00		3	8	2.00	0.82	-	-		-	-	3	0.06	
vomiting		-	-	-	-	1	2	2.00	0.00		0	0	-	-	-	-		-	-	1	0.44	
diarrhoea		-	-	-	-	0	0	0.00	0.00		0	0	-	-	-	-		-	-	0	0.10	

* p<0.002 was considered significant when adjusted for multiple testing.

# Adverse events recorded in RUNMC I and Oxford were not graded.

Unsolicited adverse events were infrequent and never severe, and all resolved spontaneously. Events included vasovagal collapse, epistaxis, flatulence, insomnia, tinnitus, hyperesthesia, psychiatric complaints associated with chloroquine [7], pleuritic chest pain, sore throat, migraine, gingivitis, palpitations, numbness in fingers, dizziness, drowsiness, photosensitivity and stiff neck.

### Laboratory safety parameters

Clinical laboratory parameters in RUNMC and USMMVP did not show significant abnormalities in hemoglobin content; parameters were not routinely available in Oxford. The majority of volunteers experienced a mild to moderate decrease in leucocyte count, none of which was severe (<1.5×10^9^/l). Thirteen of 110 volunteers (12%) showed a severe decrease in platelet count (<100×10^9^/l). All instances occurred after initiation of anti-malarial treatment, three in the delayed treatment group in RUNMC I, nine in RUNMC II and one in USMMVP. In all events platelet counts fully recovered without complications of bleeding. There were no differences in mean platelet count between the cohorts at similar timepoints (Table 1).

Volunteers with a severe decrease in platelets showed a longer period of submicroscopic parasitemia (Mann-Whitney p = 0.03), a trend towards a longer prepatent period (Mann-Whitney p = 0.079), but no higher peak parasite density (p = 0.29). The platelet nadir correlated with the pre-exposure platelet count (Spearman p = 0.005).

No abnormalities of urea and creatinine were found, but USMMVP reported six cases of hemoglobinuria and one case of proteinuria [6]. Urinary parameters were not checked in Oxford or RUNMC. Incidental increases particularly in alanine aminotransferase and aspartate aminotransferase were reported in both RUNMC and USMMVP. Severe increases (>2.5x ULN) were found in five cases, three from USMMVP (twice at day 10, once at day 14–16) and two from RUNMC II (day 16 and 17). All tests normalized at the end of the trial.

### Parasitemia by microscopy

**Figure 1:**
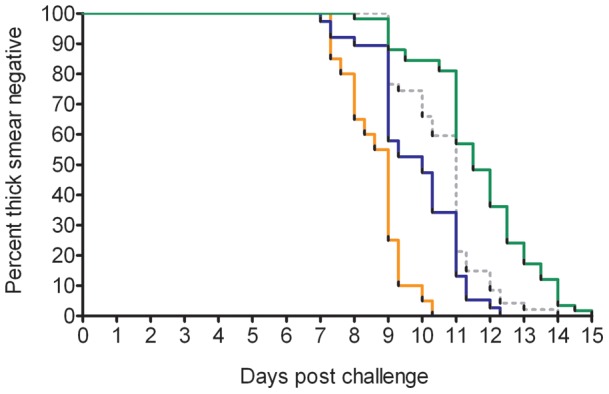
Time to microscopically detected parasitemia by cohort. Survival curve for four cohorts: RUNMC I (orange), RUNMC II (blue), USMMVP (interrupted grey line) and Oxford (green).

Prepatent period between the four cohorts was significantly different (Kruskall-Wallis p<0.001, Table 1), being longest in Oxford and shortest in RUNMC I (Figure 1). When defining prepatent period as time to one microscopically identified parasite, with adjustment for microscopic threshold of detection, the difference between prepatent period in RUNMC I and II was no longer significant (Mann-Whitney p = 0.28).

### Parasitemia by Q-PCR

Data on parasite kinetics by Q-PCR were available for all volunteers at all timepoints in RUNMC (both cohorts) and Oxford and always preceeded parasitemia by microscopy.

**Figure 2:**
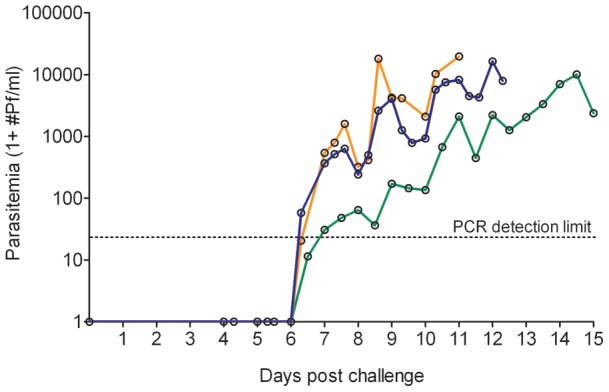
Geometric mean parasite density by Q-PCR per cohort. Three cohorts are depicted RUNMC I (orange), RUNMC II (blue) and Oxford (green).

**Figure 3:**
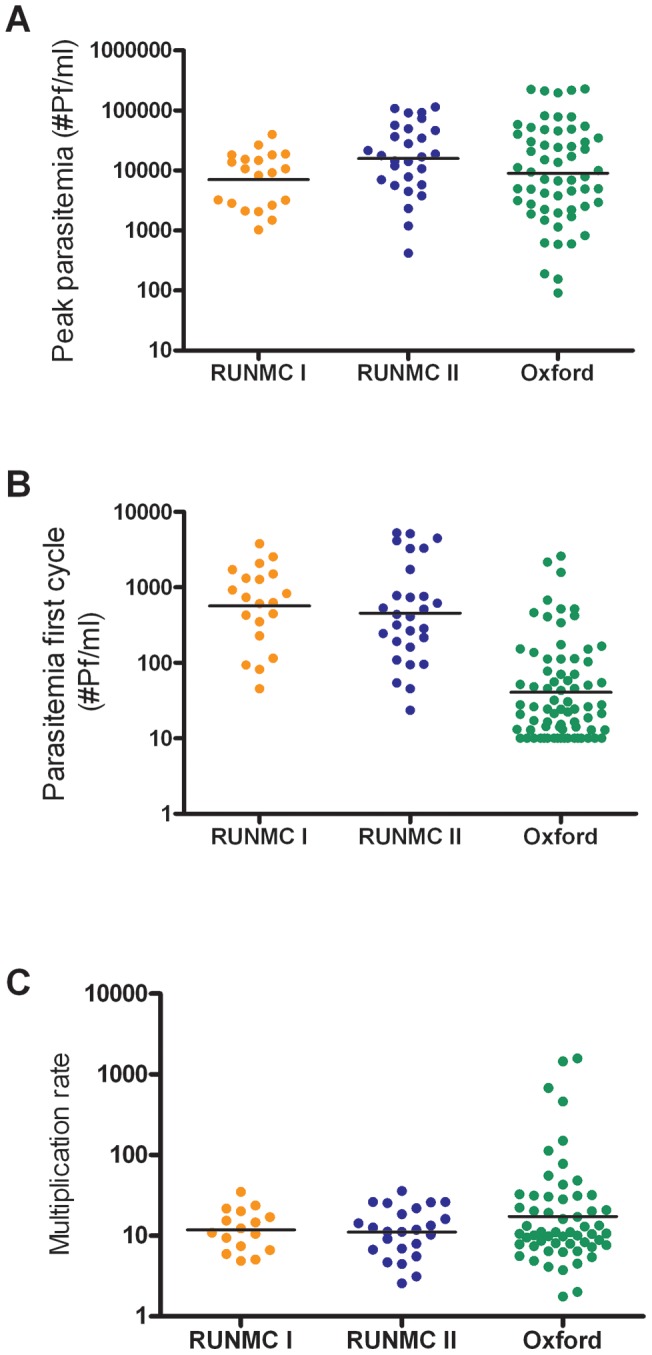
Statistics of Q-PCR parasitemia per cohort. Peak parasitemia (A, one-way ANOVA p = 0.13), geometric mean parasitemia during the first blood stage parasite multiplication cycle, day 6.5 to 8.5 (B, one-way ANOVA p<0.001) and blood stage multiplication factor (C, one-way ANOVA p = 0.97) per cohort. Individual data is plotted, lines represent geometric means. The threshold of detection of parasites by Q-PCR was 20 parasites/ml blood. Any CT value >45 was assigned a parasitemia of 10 parasites/ml.

**Figure 4:**
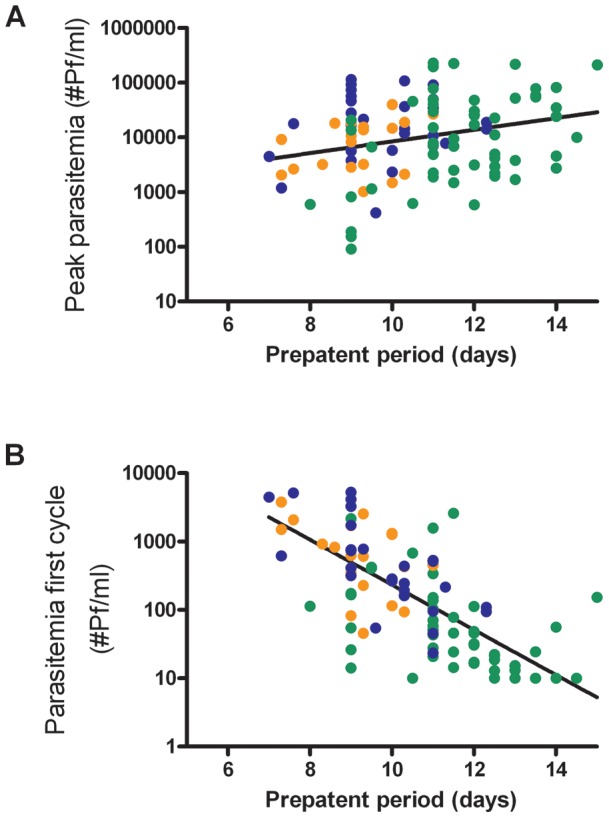
Correlation of Q-PCR parasitemia with prepatent period. Correlation between peak parasitemia (A) or geometric mean parasitemia during the first multiplication cycle from day 6.5 to day 8.5 (B) with prepatent period (R^2^ = 0.27 and −0.73, p = 0.006 and p = <0.001, respectively). Prepatent period was defined as the time between exposure to infectious mosquito bites until detection of blood stage parasites by microscopy.

Group mean parasite kinetics in RUNMC and Oxford display highly synchronized, cyclical parasite growth (Figure 2). Peak parasitemia was comparable between the cohorts despite different microscopic detection threshold (p = 0.06, Figure 3A), although there seemed to be a trend towards higher parasitemia at RUNMC after the threshold for microscopic parasite detection changed. The first blood stage parasite growth cycle was significantly lower in Oxford as compared to RUNMC (p<0.001, Table 1 and Figure 3B), whereas the blood stage multiplication rate was strikingly similar (geometric mean multiplication factor of 11.4 and 11.6 respectively, p = 0.67). This difference could not be explained by a methodological difference in Q-PCR technique, as confirmed by an exchange of samples between both institutions (data not shown). The length of the prepatent period correlated with the first blood stage growth cycle (Figure 4B) and showed a weak positive correlation with peak parasitemia (Figure 4A).

## Discussion

A comparison of clinical and parasitological data from more than ten years' experience with CHMI in RUNMC and Oxford [6], reveals significant differences in time to microscopically detected parasitemia whereas clinical symptoms are broadly similar. Although there are several methodological differences between the CHMI protocols that limit direct comparability, this difference in prepatency likely results from variation in the mean parasite burden in the first blood stage, possibly as a result of different inoculum size, whilst blood stage multiplication factors are equal.

The analysis of adverse events from 175 non-immune participants of sporozoite challenge trials shows that serious adverse events (grade 4) are rare. One serious cardiac adverse event was reported; the true nature and patho-physiological explanation of that event remains unclear [21]. Severe adverse events (grade 3) related to clinical malaria occur in up to half of the volunteers and persist for several days. We conclude that CHMI are generally safe, but may lead to severe (grade 3) symptoms, though not serious adverse events, in a significant proportion of subjects. Several precautions are taken in both institutions to ensure safety of volunteers, such as 24-hour phone access, medic-alert cards and emergency contact procedures. Nevertheless, the exposure of volunteers to the likelihood of some severe adverse events should be carefully weighed against the benefits of the information to be gained [22].

We find significant differences in frequencies of fever, fatigue, arthralgia and chills between institutions. Cohorts with a longer prepatent period or a higher peak parasitemia do not consistently show a higher frequency of adverse events. These data, combined with methodological differences in the recording of adverse events (e.g. home-monitoring of oral temperature in RUNMC), lead us to conclude that biologically relevant variation in patho-physiology between institutions seems unlikely. Nevertheless, standardized assessment of adverse events and harmonization of solicited events would advance the interpretability and comparability of CHMI in different settings worldwide.

Laboratory safety parameters show a severe decrease in platelet count (<100×10^9^/l) in at least 7.5% of volunteers, as has been studied by de Mast et al. [23], [24]. Not all centers routinely perform daily follow-up of platelets, so the actual occurrence of severe thrombocytopenia may be higher. Platelet count decreases could be predicted by baseline thrombocyte counts and the duration of blood stage parasitemia, corroborating field data where parasite density generally correlates with thrombocytopenia [25]. Although bleeding or thrombogenic complications are not reported, in trials where longer parasitemia is expected, platelet count monitoring should be considered.

An increase in threshold for microscopic detection of parasites leads to a prolonged prepatent period but not an increase in the number of adverse events, as illustrated by comparing the RUNMC I and II cohorts. Standardized reading of blood smears is thus essential to harmonize trial endpoints worldwide; recent efforts by the WHO have resulted in a proposed harmonization document (*Laurens MB, Roestenberg M, and Moorthy VS manuscript in preparation*). Variability in prepatent period among institutions, however, cannot be explained solely by microscopy methodology. A detailed analysis of parasitemia by Q-PCR revealed a ∼10 fold difference in the parasite burden during the first blood stage growth cycle. Taking into account a replication factor of approximately 10 every 48 hours, this difference accounts for a 48 hour shorter prepatent period at RUNMC. The variation in parasite load may reflect variation in liver stage development of the parasites or, alternatively, the number of inoculated parasites. The number of inoculated parasites is estimated to vary widely (ranging from 5–10 [26] to 100–300 sporozoites per bite [27], [28]). Whether the intensity of mosquito infection (e.g. sporozoite salivary gland load) or exposure time influences the number of sporozoites inoculated is controversial [29], [30]. However, also a formal relation between the number of parasites inoculated and prepatent period has never been established [31], [7], [32], [30], [28], [33]. Similarly, the role of mosquito infectivity (i.e. number of sporozoites per mosquito), parasite strain (3D7 vs NF54), exposure time (5 vs 10 minutes) or viability of inoculated sporozoites in determining the inoculated dose is unclear [31], [30]. Efforts to standardize the sporozoite dose should ideally be tested for their impact on comparability and reproducibility of CHMI. Standardization may be achieved by harmonization of mosquito breeding and feeding protocols or by needle injection of cryopreserved sporozoites. CHMI trials are underway to test the infectiousness of cryopreserved sporozoites by needle injection (NCT01086917) [34].

We show that blood stage parasite growth is cyclical and highly synchronous within and between institutions. Importantly, the duration of parasite liver stage development as well as the blood stage multiplication rate are highly reproducible. Thus vaccine efficacy can be robustly evaluated in any of the CHMI centers if a non-protected, malaria-naïve control group is included.

CHMI trials do not fully mimic conditions in endemic regions where pre-existing immunity may augment or impair vaccine efficacy. A limited number of comparisons between Phase IIa preliminary efficacy trials and Phase IIb field efficacy trials shows that results are generally in line, but more comparisons are required before definite conclusions can be drawn [2]. Another potential difference is reflected by the almost instant delivery of parasites by five infected mosquitoes, which has been considered unnatural and a stringent test for vaccine-induced immune responses [35]. However, although the frequency of infectious mosquito bites is generally lower in malaria-endemic areas, intense transmission can occur. A person may be subjected to 35–96 mosquito bites per night, and in certain areas approximately 10% of mosquitoes are infected with P*f* [36].

The present data show that CHMI can be safely conducted, but will lead to grade 3 adverse events in a proportion of volunteers. The primary parasitological outcome of such experiments is highly reproducible within institutions but may vary between trial centers. With an increasing number of CHMI centers being installed, priority should be given to initiatives to standardize challenge procedures [37]. The implementation of guidelines will enhance the comparability of CHMI; a critical and indispensable component of malaria vaccine development worldwide.
